# Successful ovulation and pregnancy with pulsatile GnRH pump therapy in a polycystic ovary syndrome (PCOS) patient: a case report

**DOI:** 10.3389/fendo.2026.1657770

**Published:** 2026-04-29

**Authors:** Shumin Wang, Ying Xiong, Lang Qin, Kehui Xu, Meng Cheng

**Affiliations:** 1 Department of Obstetrics and Gynecology, West China Second University Hospital, Sichuan University, Chengdu, China; 2 Key Laboratory of Birth Defects and Related Diseases of Women and Children, West China Second University Hospital, Sichuan University, Ministry of Education, Chengdu, China; 3 Department of Reproductive Endocrinology, West China Second University Hospital, Sichuan University, Chengdu, China

**Keywords:** case report, infertility, ovulation induction, polycystic ovary syndrome, pulsatile gonadotropin-releasing hormone pump therapy

## Abstract

Polycystic ovary syndrome (PCOS) is one of the main causes of anovulatory infertility in women of reproductive age. The main ovulation induction protocols currently used in clinical practice primarily include letrozole, clomiphene, and gonadotropins. However, some patients may exhibit drug resistance or experience ovarian hyperstimulation syndrome (OHSS). In this article, we report a case of a 28-year-old patient with PCOS who had a normal body mass index (BMI) and normal level of luteinizing hormone (LH), failed to conceive with letrozole combined with human menopausal gonadotropin (HMG) but achieved successful ovulation and pregnancy with pulsatile gonadotropin-releasing hormone (GnRH) pump therapy, without OHSS or other adverse effects. This case demonstrates the potential efficacy of pulsatile GnRH pump therapy in PCOS patients who have failed to conceive with conventional ovulation induction, suggesting its potential as a promising alternative in clinical management.

## 1 Introduction

Polycystic ovary syndrome (PCOS) is one of the most common endocrine and metabolic disorders among women of reproductive age, with epidemiological studies reporting a global prevalence of 6%–10% (1). According to the 2003 Rotterdam criteria, PCOS is confirmed when at least two of the following three features are present: oligo-ovulation or anovulation, clinical or biochemical hyperandrogenism, and polycystic ovarian morphology on ultrasound, while excluding other causes (2). Although the diagnostic criteria have been updated over the years, most recently in the 2023 International Evidence–Based Guideline (3), the core ‘two–out–of–three’ principle and the classification of phenotypes remain largely based on the Rotterdam criteria. The pathogenesis of PCOS remains incompletely understood. Most patients exhibit dysregulation of the hypothalamic-pituitary-ovarian (HPO) axis and abnormal secretion of follicle-stimulating hormone (FSH) and luteinizing hormone (LH), which subsequently leads to impaired follicular development and anovulation (4). For anovulatory PCOS patients desiring fertility, ovulation induction serves as the main treatment. Current ovulation induction options include letrozole, clomiphene, and gonadotropins. However, some PCOS patients demonstrate resistance to letrozole or clomiphene, while gonadotropin therapy increases the risks of multiple pregnancies and ovarian hyperstimulation syndrome (OHSS) (3). Therefore, there is a critical need to develop more effective and safer treatment options for ovulation induction to optimize the management of PCOS.

Gonadotropin-releasing hormone (GnRH), a decapeptide hormone secreted by the neurosecretory cells of the arcuate nucleus in the hypothalamus, is characterized by its pulsatile release. It acts directly on the adenohypophysis through the hypophyseal portal system to regulate the synthesis and secretion of FSH and LH, thereby promoting the development of the gonads and follicular growth (5). For patients with abnormal GnRH secretion, pulsatile GnRH pump therapy may serve as a potential therapeutic option. Administered via subcutaneous infusion of GnRH analogs, it mimics the endogenous pulsatile secretion of the hypothalamus, allowing precise regulation of LH and FSH release, thereby promoting dominant follicle development while reducing the risk of ovarian hyperstimulation syndrome (OHSS). However, pulsatile GnRH pump therapy is primarily used in patients with congenital hypogonadotropic hypogonadism (CHH) (6, 7), and evidence supporting its application in PCOS remains limited.

This study reports a case of successful ovulation and pregnancy achieved through pulsatile GnRH pump therapy in a PCOS patient who previously failed with letrozole combined with human menopausal gonadotropin (HMG). Notably, the patient presented with a lean PCOS phenotype, and her baseline LH level remained within the normal range. Unlike the obese PCOS phenotype, where lifestyle interventions such as diet and exercise are primary strategies to improve insulin sensitivity, these approaches often show limited efficacy in lean patients with normal metabolic profiles. In such cases, infertility may be driven by subtle neuroendocrine dysregulation. To our knowledge, reports of pulsatile GnRH pump therapy in lean PCOS patients with normal baseline LH levels remain limited. This report aims to provide preliminary clinical evidence for this therapeutic approach and highlights a potential strategy for managing infertility in this patient subgroup.

## 2 Case presentation

### 2.1 Medical history

A 28-year-old woman presented with oligomenorrhea and fertility difficulties. Her menstrual history was irregular since menarche at age 14, with cycle intervals ranging from 1 to 7 months and a duration of 5 days. She had been on long-term treatment with ethinylestradiol/cyproterone acetate (Diane-35, Bayer Weimar GmbH und Co. KG, Germany). The patient has no history of childbirth and has been trying to conceive for two years. She received two cycles of clomiphene-induced ovulation at another hospital. In the first cycle, the dosage of clomiphene citrate was 50 mg per day for 5 consecutive days. In the second cycle, the dosage was increased to 100 mg per day, and it was also administered for 5 consecutive days. No dominant follicle development was observed in either cycle. Therefore, she presented to our hospital with a desire for conception.

### 2.2 Physical examination

The patient has a body mass index (BMI) of 18.35 kg/m² with no clinical hyperandrogenism (absent hirsutism, acne, or alopecia). Gynecological examination shows normal external genitalia, vagina, and cervix, with an anteverted uterus of normal size.

### 2.3 Auxiliary examination

Transvaginal ultrasound revealed normal uterine dimensions and bilateral polycystic ovarian morphology (PCOM). Each ovary contains more than 12 follicles measuring 2–9 mm in diameter. Hormone test on the second day of the menstrual cycle and laboratory evaluations demonstrated: Follicle-stimulating hormone (FSH) 6.96 IU/L, Luteinizing hormone (LH) 6.46 IU/L, Estradiol (E_2_) 32.5 pg/mL, Progesterone (P) <0.16 ng/mL, Prolactin (PRL) 6.84 ng/mL, Total testosterone (TT) 0.13 ng/mL, Dehydroepiandrosterone sulfate (DHEA-S) 131 μg/dL, Androstenedione: 1.34 ng/mL, Anti-Müllerian hormone (AMH) 5.05 ng/mL, Thyroid-Stimulating Hormone (TSH) 1.363 mIU/L, Fasting Blood Glucose (FBG) 4.23 mmol/L, Fasting Insulin (INS0) 3.05 μIU/mL, Homeostatic Model Assessment of Insulin Resistance (HOMA-IR) 0.57.

### 2.4 Diagnosis

Based on the presence of oligomenorrhea and PCOM, with the exclusion of other endocrine disorders, the patient was diagnosed with PCOS according to the Rotterdam criteria and the 2023 International Evidence-based Guideline (2, 3).

### 2.5 Traditional ovulation induction therapy

The patient underwent four cycles of letrozole (Jiangsu Hengrui Medicine Co., Ltd, China) combined with HMG (Livzon Pharmaceutical Group Inc., China) stimulation. In the first treatment cycle, the patient received letrozole 2.5 mg per day for 5 days and total HMG 375 IU. In the second treatment cycle, the dosage was adjusted to letrozole 5 mg per day for 5 days and total HMG 375 IU. The third treatment cycle involved letrozole 3.75 mg per day for 5 days and total HMG 225 IU, while the fourth cycle used letrozole 5 mg per day for 5 days and total HMG 150 IU. Ovulation occurred in the first cycle without pregnancy, and the subsequent three cycles were cancelled due to excessive multifollicular growth. The follicular response with letrozole combined with HMG is shown in **Table 1**.

**Table 1:** The follicular response with letrozole combined with HMG.

Treatment cycle	LE	Response to LE (Maximum follicle size, mm)	Interval time from LE to HMG (day)	HMG	Ovulation induction result	Trigger medicine	Pregnancy outcome
Cycle 1	2.5 mg qd for 5 days	9.0	4	75 IU Qod for 5 days	A single dominant follicle formation	rhCG 250 ug	Failed
Cycle 2	5 mg qd for 5 days	10.0	3	75 IU Qod for 5 days	Multiple follicular development*	Cancellation	N/A
Cycle 3	3.75 mg qd for 5 days	9.5	3	75 IU Qod for 3 days	Multiple follicular development*	Cancellation	N/A
Cycle 4	5 mg qd for 5 days	8.0	1	75 IU Qod for 2 days	Multiple follicular development*	Cancellation	N/A

LE, letrozole; HMG, human menopausal gonadotropin; rhCG, recombinant human chorionic gonadotropin.

* Multiple follicular development means there are more than 3 follicles larger than 15 mm in diameter.

N/A, not applicable.

### 2.6 Pulsatile GnRH pump therapy

Due to a failure to achieve pregnancy with letrozole combined with gonadotropins, approved by the Ethics Committee of West China Second University Hospital (Approval No. 2020-041), the patient was provided informed consent and accepted pulsatile GnRH pump therapy (subcutaneous pulse injection, gonadorelin, Fengyuan Pharmaceutical Co., Anhui Province, China). The patient’s last menstrual period was August 5, 2024, and the pulsatile GnRH pump therapy was initiated on day 3 of the menstrual cycle. The initial regimen was set at 10 μg per pulse every 90 minutes, with subsequent dose and frequency adjustments based on sex hormone and follicular tracking. On day 10 of the menstrual cycle, LH levels increased significantly, and the frequency of the GnRH pump was adjusted to pulse every 120 minutes. On day 17 of the menstrual cycle, the follicles were developing slowly, the dose of the GnRH pump was increased to 15 μg, and since then, the follicles have developed well. On day 24 of the menstrual cycle, ovulation was confirmed, but no pregnancy occurred.

The patient experienced the second treatment cycle with a GnRH regimen maintained at 15 μg per pulse every 120 minutes. Hormonal evaluation on cycle day 8 revealed FSH 4.0 IU/L, LH 12.8 IU/L, E_2_ 313.2 pg/mL, P 0.26 ng/mL. Ultrasound monitoring identified a dominant follicle measuring 19 × 18 mm in the left ovary. Ovulation was triggered using 250 μg of recombinant human chorionic gonadotropin (Merck Serono S.p.A., Italy). Pulsatile GnRH pump therapy was continued, and luteal phase support was initiated using dydrogesterone (Abbott Biologicals B.V., The Netherlands). On cycle day 28 (20 days post-trigger), human chorionic gonadotropin level was 1260.3 IU/L and progesterone level was 15.29 ng/mL, indicating biochemical pregnancy. Then, the GnRH pump was removed, and luteal phase support was continued with dydrogesterone. On October 31, 2024 (41 days post-trigger), a transvaginal ultrasound confirmed intrauterine pregnancy with fetal cardiac activity. The medication protocols and changes in follicular development and hormone levels are shown in **Table 2**.

**Table 2:** The medication protocols and changes in follicular development and hormone levels in the Pulsatile GnRH pump therapy Cycles.

Treatment cycle	Cycle day	Gonadorelin dose/frequency	Other drug	Maximum follicle size (mm)	Endometrial thickness (mm)	FSH (IU/L)	LH (IU/L)	E_2_ (pg/mL)	P (ng/mL)	HCG (IU/L)
The first cycle	Day 3	10 μg per pulse every 90 minutes	None	5.0	4.0	1.9	3.9	19.0	0.21	N/A
	Day 10	10 μg per pulse every 120 minutes	None	9.5	6.0	5.0	23.8	42.7	0.31	N/A
	Day 17	15 μg per pulse every 120 minutes	None	11.5	6.6	5.2	13.1	95.3	0.26	N/A
	Day 21	15 μg per pulse every 120 minutes	None	18.5	8.8	11.7	103.3	283.1	0.48	N/A
	Day 24	15 μg per pulse every 120 minutes	Dydrogesterone	11.5	10.0	N/A	N/A	N/A	N/A	N/A
The second cycle	Day 8	15 μg per pulse every 120 minutes	rhCG	18.5	8.0	4.0	12.8	313.2	0.26	N/A
	Day 12	15 μg per pulse every 120 minutes	Dydrogesterone	10.5	12.0	N/A	N/A	N/A	N/A	N/A
	Day 28	Gonadorelin discontinuation	Dydrogesterone	N/A	N/A	N/A	N/A	523.5	15.29	1260.3

FSH, Follicle-stimulating hormone; LH, Luteinizing hormone; E_2_, Estradiol; P, Progesterone; HCG, human chorionic gonadotropin; rhCG, recombinant human chorionic gonadotropin.

N/A, not applicable.

### 2.7 Pregnancy follow-up

The patient received regular prenatal care throughout the pregnancy without complications such as gestational diabetes mellitus, hypertensive disorders, or cardiac disease. The fetal development was in good condition. The color Doppler ultrasound revealed vasa previa at 26 + 3 weeks of gestation, and the patient underwent a cesarean section at 35 + 6 weeks of gestation due to vasa previa on May 22, 2025, with good maternal and neonatal outcomes.

## 3 Discussion

PCOS is the most common endocrine cause of anovulatory infertility among reproductive-aged women (8). A feature of PCOS is abnormally increased GnRH pulse frequency, which leads to excessive LH secretion and relatively insufficient FSH production. This neuroendocrine dysregulation stimulates the ovaries to over-secrete androgens, inhibiting follicle development and maturation, and finally leads to anovulatory infertility (9). For PCOS patients seeking fertility, lifestyle management combined with ovulation-inducing medications such as letrozole, clomiphene citrate, or gonadotropins can often restore ovulation. According to the 2023 guidelines, letrozole is recommended as the first-line agent for ovulation induction in patients with PCOS (3). Letrozole acts by inhibiting the conversion of androstenedione and testosterone into estrogen, thereby reducing the negative feedback on the hypothalamic–pituitary–ovarian (HPO) axis (10). For patients who respond poorly to letrozole, gonadotropins may be considered as a second-line therapeutic option to promote follicular development; however, this approach is associated with an increased risk of multiple follicular development and ovarian hyperstimulation syndrome (OHSS) (3). In addition to pharmacological interventions, laparoscopic ovarian drilling (LOD) may also be considered a second-line treatment for anovulatory infertile women with PCOS who are resistant to clomiphene citrate and have no other infertility factors. LOD aims to reduce ovarian androgen production by selectively destroying portions of the ovarian stroma, thereby potentially restoring spontaneous ovulation. However, due to its invasive nature and potential complications, such as ovarian atrophy or adhesion formation, its clinical use is generally limited.

In the natural menstrual cycle, rising follicular-phase estrogen suppresses FSH via negative feedback, ensuring single dominant follicle selection and atresia of the remaining follicles (11). In contrast, exogenous gonadotropins bypass the HPO axis feedback and directly stimulate ovarian follicles, allowing multiple follicles to escape atresia and resulting in multifollicular development and excessive estrogen secretion (12, 13). Meanwhile, the secretion of vascular endothelial growth factor (VEGF) increases, which enhances systemic capillary permeability and may ultimately induce OHSS (14). This series of pathological processes is particularly prominent in patients with PCOS (15). In our case, the patient exhibited multifollicular growth with letrozole plus gonadotropins, but achieved monofollicular development and pregnancy with pulsatile GnRH pump therapy. Compared with gonadotropin therapy, pulsatile GnRH pump therapy has two main advantages. First, it preserves the integrity of the HPO axis feedback system by mimicking the physiological pattern of GnRH secretion. Second, it allows dynamic adjustment of GnRH pulse dose and frequency according to sex hormone levels and follicular development. Increased GnRH pulse frequency preferentially stimulates LH secretion, and decreased GnRH pulse frequency enhances FSH production (16). These features result in more physiological FSH and LH secretion patterns, thereby reducing the risks of multiple pregnancy and ovarian hyperstimulation (17, 18).

It should also be noted that PCOS is not a single disease entity but a highly heterogeneous clinical syndrome. One important aspect of this heterogeneity is the distinction between obese and lean phenotypes. Obese PCOS is typically characterized by obesity and pronounced insulin resistance. In contrast, lean PCOS patients usually present with normal or low BMI and relatively mild metabolic disturbances, while neuroendocrine dysregulation tends to play a more prominent role. In many lean PCOS patients, dysregulation of the hypothalamic GnRH pulse generator leads to an increased GnRH pulse frequency, which often results in elevated LH secretion (19). In our case, the patient had a normal BMI of 18.35 kg/m² and was therefore classified as lean PCOS; however, her baseline LH level remained within the normal range. Previous studies have shown that LH secretion during the reproductive cycle is influenced not only by the frequency of GnRH pulses but also by their amplitude (20, 21). It is possible that this patient exhibited accelerated GnRH pulsatility with relatively low amplitude or irregular rhythmicity, resulting in a normal circulating LH level. In this context, pulsatile GnRH pump therapy may restore a more physiological pattern of GnRH pulses by externally delivering controlled GnRH pulses and dynamically adjusting the stimulation parameters, thereby effectively “resetting” the disrupted neuroendocrine rhythm. The successful pregnancy and live birth achieved in this patient further support the therapeutic value of this approach in lean PCOS patients with normal LH levels.

Early studies have also explored the use of pulsatile GnRH pump therapy in patients with PCOS. In a study published in 1989, Marco Filicori et al. reported that pulsatile GnRH pump therapy alone showed limited efficacy in inducing ovulation in patients with PCOS, but ovulation rates increased significantly following pretreatment with a GnRH agonist (22). In 1994, the same research group expanded their sample size to 172 cycles and further confirmed this conclusion (23). Pretreatment with a GnRH agonist can downregulate the HPO axis, thereby correcting abnormal endocrine patterns and restoring hormone secretion toward physiological levels. In contrast, our patient had normal baseline LH levels, so GnRH agonist pretreatment was unnecessary, and the HPO axis responded well to pulsatile GnRH pump therapy. She initially received 10 μg per pulse every 90 minutes, which increased LH levels, accompanied by impaired follicular development. After adjustment to 15 μg per pulse every 120 minutes, LH levels decreased, follicular maturation improved, and pregnancy was achieved in the second cycle. Similarly, previous case reports have documented successful pregnancies in PCOS patients—including obese and lean individuals, and those with insulin resistance treated with metformin—following pulsatile GnRH pump therapy (24–26). These findings suggest that HPO axis regulation is central to ovulation induction in PCOS, regardless of other metabolic or endocrine factors.

Although pulsatile GnRH pump therapy has demonstrated clinical efficacy in patients with PCOS, standardized administration protocols have yet to be established. Research remains limited, with many studies conducted decades ago, and reported GnRH dosages and pulse frequencies vary. Graña-Barcia et al. reported that subcutaneous pulsatile GnRH with 20 μg per pulse every 90 minutes significantly reduced serum FSH and LH levels (27). Seang-Lintan et al. achieved a 50% ovulation rate among clomiphene-resistant PCOS women using 15 μg per pulse every 90 minutes (28). In addition, comparative research evaluating pulsatile GnRH pump therapy (15-20 μg per pulse every 90 minutes) versus combined gonadotropins (rFSH+rLH) in hypothalamic amenorrhea/PCOS patients revealed a higher clinical pregnancy rate with pulsatile GnRH pump therapy (46.6% vs. 0%) (29). In our case, therapy was initially administered at 10 μg per pulse every 90 minutes and subsequently adjusted to 15 μg per pulse every 120 minutes, which led to a successful pregnancy. Thus, further studies are needed to standardize the clinical use of GnRH pumps in patients with PCOS. In addition, the relatively high cost and the technical requirements may limit its accessibility, making pulsatile GnRH pump therapy generally unsuitable as a first-line option. However, it remains a less invasive and potentially more economical alternative to second-line intervention, laparoscopic ovarian drilling.

## 4 Conclusion

In summary, this case demonstrates that pulsatile GnRH pump therapy can effectively induce mono-follicular ovulation and lead to a successful live birth in a patient with PCOS. This therapeutic approach may represent a potential management option for the management of lean PCOS patients with normal baseline LH levels. It also suggests that it could serve as a potential second-line treatment option for patients who fail to ovulate or conceive with conventional ovulation induction. However, given that this is a single case report, further large-scale prospective studies are needed to explore the application of this therapy in the management of PCOS.

## 5 Patient perspective

“After experiencing so many failed ovulation induction cycles, I felt extremely disappointed and frustrated. Upon learning about the pulsatile GnRH pump therapy from my doctor, I decided to give it a try. Now, I am very happy and fortunate to have a healthy baby. I hope this treatment method can benefit more patients like me.”

## Data Availability

The original contributions presented in the study are included in the article. Further inquiries can be directed to the corresponding author.
